# Research into the Causes of Venom-Induced Mortality and Morbidity Identifies New Therapeutic Opportunities

**DOI:** 10.4269/ajtmh.17-0877

**Published:** 2019-01-21

**Authors:** Kesturu S. Girish, Gajanan D. Katkar, Robert A. Harrison, Kempaiah Kemparaju

**Affiliations:** 1 Department of Studies in Biochemistry, University of Mysore, Mysuru, India;; 2 Department of Studies and Research in Biochemistry, Tumkur University, Tumakuru, India;; 3 Cardiovascular Research Lab, Institute of Biomedical Sciences, Academia Sinica, Taipei, Taiwan;; 4 Centre for Snakebite Research and Interventions, Liverpool School of Tropical Medicine, Liverpool, United Kingdom

## Abstract

Snakebite primarily affects rural subsistent farming populations in underdeveloped and developing nations. The annual number of deaths (100,000) and physical disabilities (400,000) of snakebite victims is a societal tragedy that poses a significant added socioeconomic burden to the society. Antivenom therapy is the treatment of choice for snakebite but, as testified by the continuing high rates of mortality and morbidity, too many rural tropical snakebite victims fail to access effective treatment. Here, we advocate for more basic research to better understand the pathogenesis of systemic and local envenoming and describe how research outcomes can identify novel snakebite therapeutic strategies with the potential to be more accessible and affordable to victims than current treatment.

## INTRODUCTION

Snakebite is a daily, domestic, occupational, and environmental hazard in the tropical countries of Asia, Africa, and Latin America.^1–4^ Of the 1.8–2.7 million people bitten annually by snakes, approximately 81,000–138,000 victims die and about 400,000 survivors suffer from permanent physical disabilities.^5^ The snakebite mortality and morbidity figures may be a substantial underestimate because many, perhaps most, victims do not access hospital care and their deaths and disabilities are therefore not recorded. For example, a recent community-based study^1^ undertaken throughout India (as part of the million deaths project) identified that snake envenoming kills 46,000 victims a year—significantly more than had been estimated previously. Thus, the continuing high rates of mortality and morbidity indicate the scale of the problem. Despite this being a global threat, snakebite remains more of a poor man’s problem; furthermore, unlike infectious diseases to which the world pays greater attention, the noninfectious nature of snakebite could also be the reason for it’s negligence. The epidemiological data reveals that children and adults, mostly the breadwinners of the families, from the weaker socioeconomic background engaged in subsistence farming are the most vulnerable to snakebite.^5^ The affected families thus suffer from sustained socioeconomic crisis including child development and income loss. There are studies reported that the contribution of snakebite to disease burden is half the disease burden of HIV/AIDS in developing and underdeveloped countries.^6^ This disease burden is likely to be replicated in many tropical and subtropical countries and underscores the need for immediate attention to deliver more accurate medical care, including sensitizing national and international health policy-making.

Research into antivenom production for snakebite treatment has a long history, starting with Albert Calmette in 1894 who pioneered anti-snake venom serum for snakebite victims.^7,8^ Since then, intravenous infusion of antivenom remains an exclusive therapy; however, there have been innovations over the past century. These include purifying IgG fractions from the serum of various animals (horse, camel, sheep and donkey) immunized with venom.^9,10^ Furthermore, researchers in the past have also been motivated to produce therapeutic F(ab)_2_ and Fab fragments from anti-snake venom IgG through limited proteolysis.^11–13^ This was to reduce the risk of serum sickness due to overdosage of antiserum and to augment the neutralizing efficacy by increasing the number of paratopes per unit mass of antivenom. Presently, there are more than 45 antivenom-manufacturing companies using these protocols to produce a number of brands of IgG, F(ab′)_2_, or Fab antivenom for global use.^14–19^

### Antivenom therapy: Factors reducing the efficacy.

Snakebite is an example of a simultaneous potential lethal assault on vital systems such as nervous, muscular, and circulatory systems. These systems are functionally interdependent such that the effect on one system can affect the others, and thus, it is a precarious medical emergency. Despite advancement in snake venom research, there are very limited treatment options, except for the usage of antivenom therapy to save snakebite victims, including the lack of life saving first-aid protocols. Although antivenom remains the sole therapy as of now, the global high rates of mortality and morbidity reported by various studies underscore the serious drawbacks of the antivenom therapy.^20–22^ Three factors have been considered to critically affect the efficacy and success rate of the antivenom therapy: first, the delay between the time of the bite and the start of antivenom therapy (these have a reciprocal relationship). In most affected nations, victims are transported long distances from remote places to health-care centers. Furthermore, scarcity of antivenom therapy even at tertiary health-care centers is also a matter of concern, thus extending the delay. Second is the polyvalent nature of the therapeutic antivenom: for example, in India, polyvalent antivenom (prepared against the venoms of “big four” snakes: cobra, krait, Russell’s viper, and saw-scaled viper) is the only antivenom in use for the treatment. Thus, the efficacy of the therapy against the specific case of envenomation is likely to be reduced by 1/4. Third is the venom variability, which is considered at different levels, such as inter-family, inter-genus, inter-species and subspecies, and within species.^23–27^ Of these, the variability within the species is considered to be perilous, especially variability due to the varied geographic distribution of snake species. Nevertheless, it is also important to realize the fact that several studies endorse the venom variability within the species because of age, season, gender, and diet variations in addition to geographic variations.^23,25^ Thus, venom variability appears to be an innate character and could be controlled by multiple factors regulating through epigenetic and other modifications. To tackle this, in a country like India several studies strongly recommend the use of region specific antivenom.^28^

Although antivenom therapy saves the lives of hundreds and thousands of snakebite victims, the high rate of mortality is a strong indicator of underlying deficiencies. Furthermore, the therapy is unsuccessful against the sustained tissue necrosis induced at the bite site by the vipers and some cobras.^4^ Thus, under critical circumstances, continued tissue necrosis may only be prevented through amputation of the affected limb; this results in permanent disability or disfigurement. Thus, achieving successful treatment against the sustained tissue necrosis is challenging and still remains a grey area. Therefore, it is important to evaluate options for the existing antivenom therapy so that an effective therapy that would reduce the rates of both mortality and morbidity may be achieved. Here, we describe efforts to identify the mechanisms of these pathologies and how this improved understanding leads to the novel potential therapies, including those potentially suitable as “first response” measures.

### The pathogenesis and reversal of snake venom–induced local tissue destruction.

**Figure 1.**
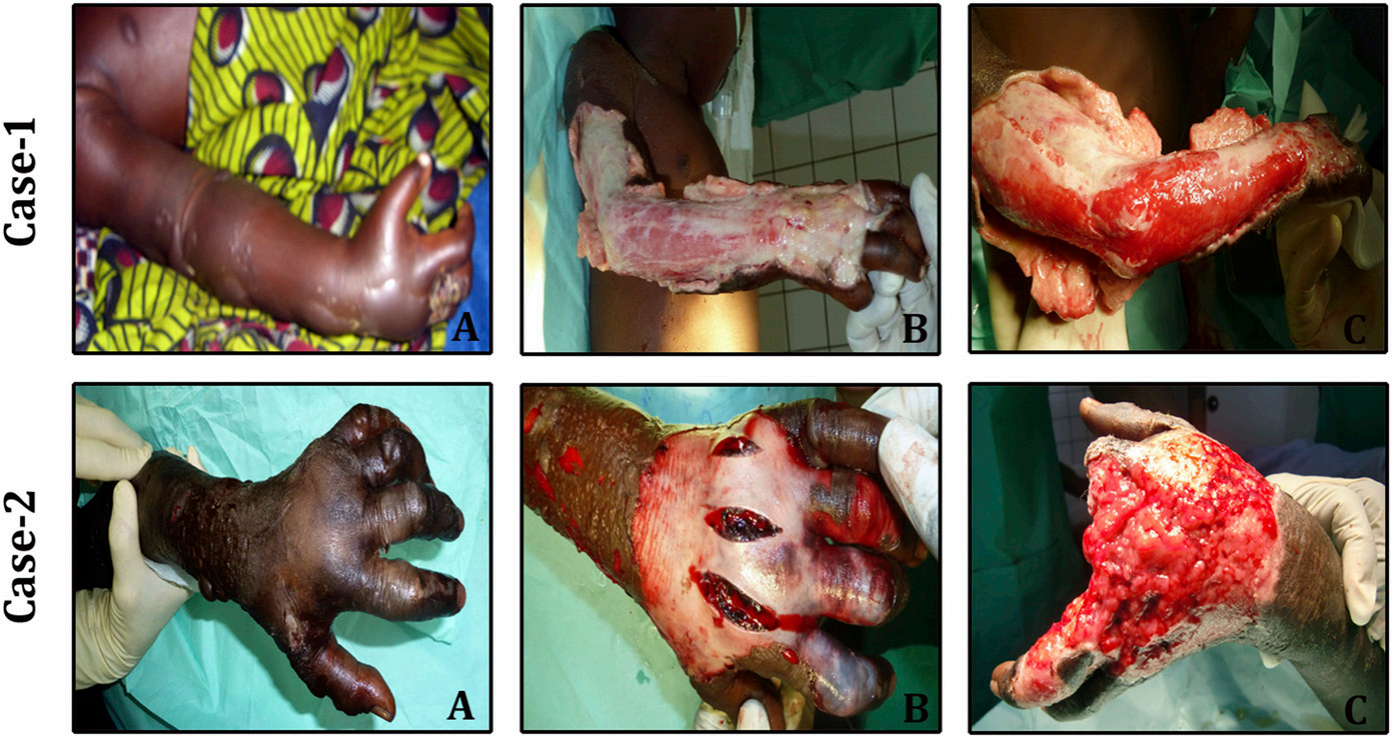
The pictures represent the gravity of snakebite-induced sustained tissue decay at the bite site. Case 1: (**A**) initial examination showed that the right hand and forearm were swollen, (**B**) initial perioperative observations, and (**C**) after wound cleaning. Case 2: (**A**) initial aspect of the left hand, (**B**) fasciotomy, and (**C**) clean aspect of the hand, after eight surgeries. Figures reprinted from Ref. 21. Copyright by CEVAP/UNESP. Reprinted with permission. This figure appears in color at www.ajtmh.org.

Envenoming by many vipers and some cobras can cause profound local tissue destruction at the bite site. Figure 1 illustrates the impact of the fearful, debilitating, tissue-destructive effects of envenoming by the West African saw-scaled viper *Echis ocellatus*.^21^ Because antivenom is ineffective in treating this condition (unless it is given very soon after the bite, which is rare in rural remote regions at greatest risk), surgical debridement or amputation is required to manage this pathology. This dreadful event remained unaddressed all these years as there were no suitable models to investigate the molecular mechanism. Our laboratory has established a mouse-tail model and investigated the inflammatory response to *Echis carinatus* (Indian saw-scaled viper) venom.

We have reported^29^ that *E. carinatus* venom induces a strong inflammatory response at the injection site starting with an influx of neutrophils (polymorphonuclear leukocytes). They are the first line of defense cells and major phagocytes of innate immunity. As an ultimate defense, neutrophils commit suicide, resulting in the release of their de-condensed DNA fibers/chromatin, which form structures called neutrophil extracellular traps (NETs) by a process called NETosis.^30–34^ We showed that *E. carinatus* venom readily elicits intense and stable NETs at the injection site through a mechanism dependent on reactive oxygen species (ROS). The extruded DNA fibers/chromatin (NETs) from hundreds and thousands of neutrophils form a physical plug in the blood vessels that 1) prevents blood flow to tail tissue distal from the venom injection site, 2) sequesters venom toxins in that site through ionic interactions, and 3) causes accumulation of toxic metabolites, and hypoxia. Furthermore, sequestration of venom toxins, such as the extracellular matrix (ECM)–degrading hemorrhagic snake venom metalloproteases (SVMPs), resulting in the degradation of blood vessels and surrounding tissues. Together, these sustained effects resulted in tail tissue necrosis.^29^

**Figure 2.**
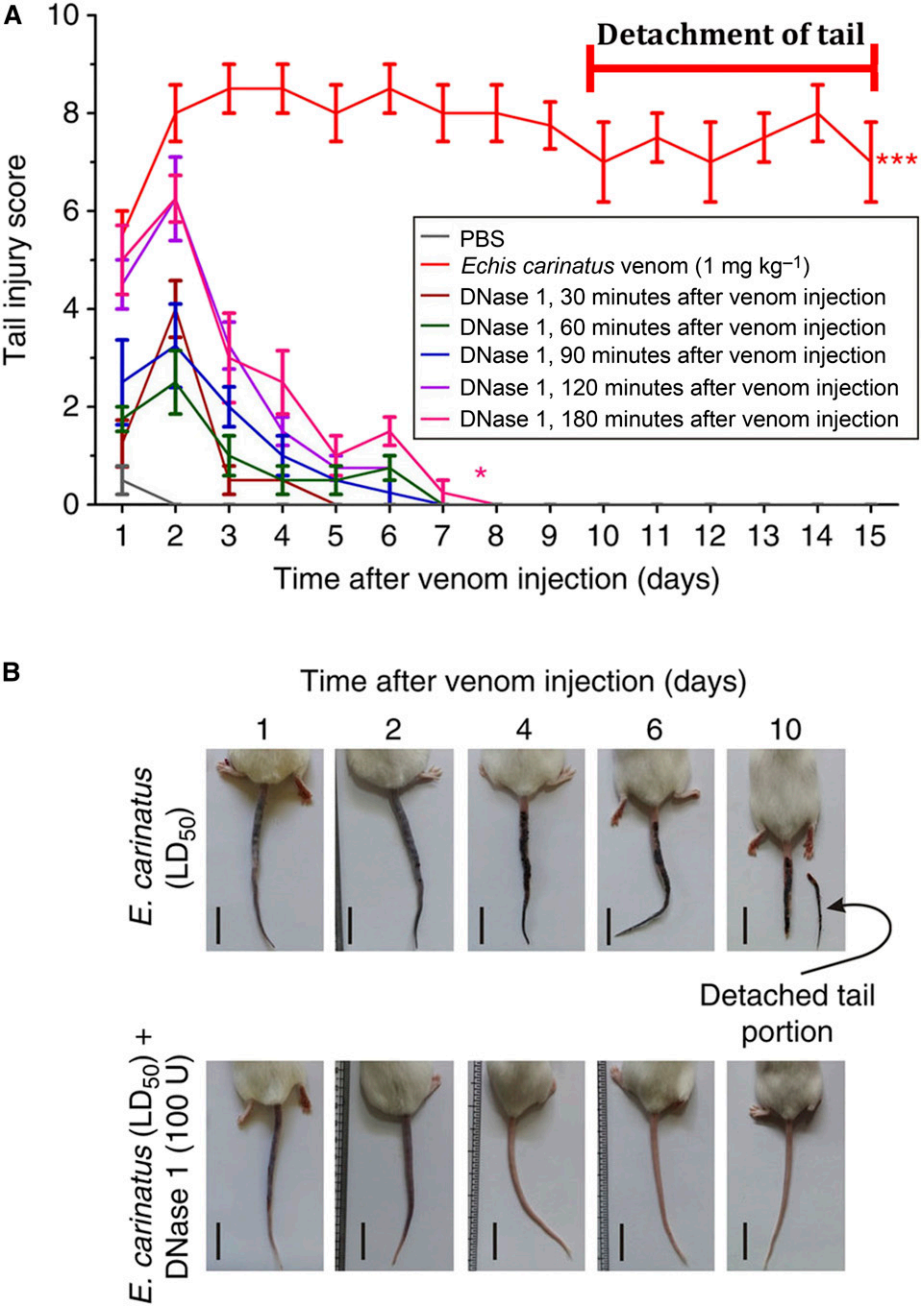
Deoxyribonuclease (DNase) 1 treatment prevents *Echis carinatus* venom-induced tissue destruction in the challenge study. (**A**) The graph represents the continued tail injury score in *E. carinatus* venom (LD_50_)-injected mouse tails (red line), whereas the administration of 100 U DNase 1 at various times (30–180 minutes post-venom injection) decreased the tail injury score. PBS = Posphate buffer saline. (**B**) Representative photographs of mice taken on different days after injection. The mice were injected with *E. carinatus* venom (LD_50_; top row) or co-injected with *E. carinatus* venom (LD_50_) and DNase 1 (100 U; bottom row); the mice in the latter group recovered, and normal tail morphology was restored from day 4 onward (bottom, third). Scale bars, 2 cm (*n* = 10). Figures reprinted from Ref. 29 with permission. This figure appears in color at www.ajtmh.org.

We reasoned that dismantling the DNA fibers/NETs should restore blood flow before the death of cells and tissues, and thereby prevent the onset of necrosis. Indeed, injection of deoxyribonuclease 1 (DNase 1) even 3 hours after the venom injection to the same site prevented necrosis (Figure 2) and enabled full physiological recovery of tail tissue.^29^

We have extended our research into the mechanisms of venom-induced tissue destruction. Neutrophils, monocytes (peripheral blood mononuclear cells), and platelets are the key players of the inflammatory response and wound-healing process and express transmembrane surface receptors for collagen, including integrin α2β1, glycoprotein VI, and discoidin domain receptor 1. These receptors regulate a spectrum of cellular functions including adhesion, migration, differentiation, hemostasis, and immune functions.^35–41^ Peripheral blood mononuclear cells also express the CX3 chemokine receptor 1, which is essential for angiogenesis and wound-healing processes.^40^ We demonstrated that 1) *E. carinatus* venom SVMPs, in addition to destroying the ECM, also degrade these receptors, resulting in the inhibition of the adhesion of inflammatory cells and hence inhibition of angiogenesis and wound-healing events, and 2) these venom-induced effects can be reversed by a lupeol-derivative treatment.^41^

These studies demonstrate the value of research into the mechanisms of venom-induced tissue necrosis and indicate that surface application of SVMP inhibitors is likely to enhance angiogenesis and wound-healing processes, and injection of DNase 1 to break down NETs could be an effective treatment of snake venom-induced tissue necrosis.

### The pathogenesis and reversal of venom-induced systemic toxicity with “first-response” treatment at the community level.

Lack of adequate health-care facilities in the underdeveloped and developing countries leads to delayed treatments after a snakebite. We reasoned that the delay in snakebite treatment may cause additional pathologies related to the acute phase response of neutrophils and other circulating blood cells associated with hypoxia. We also sought to identify a treatment that could be safely applied in the community to reduce/delay the severity of systemic snake venom pathology.

Neutrophils and platelets are abundant but short-lived and serve as a rich source of ROS, and therefore cause a significant risk of systemic oxidative stress. In addition to damaging cells, tissues, and organs, ROS also affect the gas transport system of red blood cells (RBCs). The ROS-mediated oxidation of Hb (ferrous-Fe^+2^) to MetHb (ferric-Fe^+3^) results in severe methemoglobinemia, the lack of oxygen and H^+^ binding and transport abilities. Snake envenoming is known to activate neutrophils, and we, therefore, investigated whether envenoming results in systemic oxidative stress and perhaps hypoxia.^42^

We demonstrated that the venom from the Indian cobra (*Naja naja*) induced a massive oxidative stress, an important, previously unrecognized toxic property of snake venom. Strikingly, although antivenom neutralized much of the venom-induced neurotoxicity, myotoxicity, and hemotoxicity, it did not neutralize venom-induced oxidative stress. In fact, antivenom at higher doses induced mild oxidative stress in mice.^43–46^

MetHb is itself a prooxidant molecule and, therefore, can augment the magnitude of MetHb formation and hence hypoxia. Furthermore, during methemoglobinemia, RBCs tend to form aggregates and become vulnerable to lysis, which to the causes additional physiological stress. Thus, during hypoxia, there will be a gradual dip in the pH due to the accumulation of H_2_CO_3_ (CO_2_ equivalent) and toxic metabolites in the systemic compartment. Reactive oxygen species also inhibit energy (adenosine triphosphate [ATP]) production by inhibiting electron transport chain.^47,48^ Thus, venom-induced oxidative stress will push the physiological system into a dangerous state of energy depletion and fatal hypoxia. The brain is likely the organ at the greatest risk because it is strictly aerobic and thought to use about 10 times more energy per unit mass than other organs.^49^ Contractile organs such as lungs, heart, and diaphragm also demand a continuous supply of energy and hence, they also are at particular risk of the effects of oxidative stress and hypoxia.

**Figure 3.**
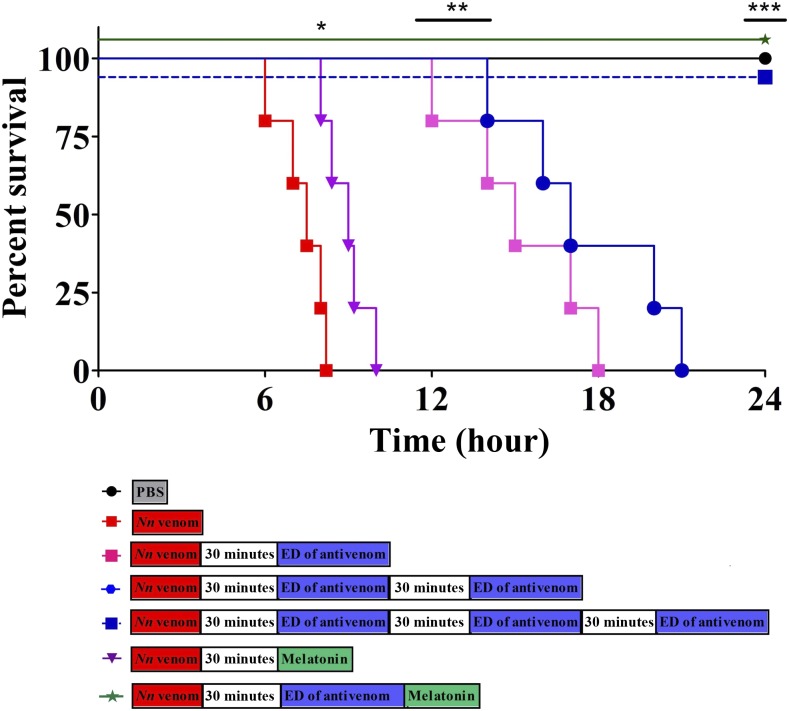
Effect of melatonin and antivenom on *Naja naja* venom–induced lethality in an in vivo challenge study. Melatonin and antivenom were administered (i.v.) independently, and in combination at various time intervals after *Nn* venom injection (i.p.). Represent the Kaplan–Meier survival analysis. *P* value was calculated using the log rank (Mantel–Cox) test. *P < 0.05, **P < 0.01, and ***P < 0.001. *Significant compared with *Nn* venom–injected group. Figure reprinted from Ref. 42 with permission. This figure appears in color at www.ajtmh.org.

This led us to investigate modalities to prevent venom-induced hypoxia, and we rationalized that protecting Hb in its ferrous-Fe^+2^ form was the key objective. We preclinically demonstrated, again for the first time, that the antioxidant melatonin not only reduced venom-induced oxidative stress but also increased the survival rate of envenomed mice and significantly reduced the neutralizing dose of antivenom.^42^ Figure 3 demonstrates that none of the mice survived after the cobra venom injection, although three effective doses (EDs; it is the amount of antivenom that neutralizes venom activity in vitro) of antivenom were needed to neutralize the lethal effect of cobra venom. Interestingly, all mice injected with venom survived after the administration of melatonin along with one ED of antivenom (Figure 3).^42^ Our finding suggests that the antioxidant administration could be used as a first-aid therapy to protect victims from developing a dangerous hypoxic state.^42^ Melatonin is safe and penetrates into the blood and cells quickly, and its metabolites (kynuramines) also function as effective antioxidants. Moreover, all these molecules are safe and readily cleared from the systemic circulation through urine. Diffusion of venom toxins into the blood alters the hemostasis because of their procoagulant or anticoagulant properties. Therefore, replacing the victim’s venom-contaminated blood with normal blood might restore the altered hemostasis and oxygen level, and reduce the systemic toxicities of snakebite victims.^50^ Blood transfusion could be an effective treatment where antivenom is inadequate. Fadare and Afolabi have successfully shown that the whole blood transfusion could be an alternative treatment for the management of snakebite in resource-challenged areas.^50^ However, detailed investigations are needed to validate the outcome.

## CONCLUSION

In conclusion, we have demonstrated that the fundamental research into the pathogenesis of snake venom-induced pathology can yield important new data that have the potential to lead to new treatment strategies—which need to be verified at the clinical level. Thus, injection of DNase 1 and application of SVMP inhibitors at the bite site could prevent venom-induced tissue necrosis. We also suggest that the administration of melatonin tablets at rural first-aid centers may prevent venom-induced hypoxic pathology while patients are transported to hospital for antivenom treatment. We hope that these discoveries offer affordable, more culturally appropriate options to improve the management of snakebite mortality and morbidity. We also illustrate the benefit of basic research and urge similar efforts by the scientific community, and investment in this research by the governing bodies of the affected nations.
